# Supplementary material for: Setd2 supports GATA3+ST2+ thymic-derived Treg cells and suppresses intestinal inflammation
Source: Nat Commun. 2022 Dec 3;13:7468. doi: 10.1038/s41467-022-35250-0 (PMC9719510; doi:10.1038/s41467-022-35250-0)

Supplementary Information

Setd2 supports GATA3$^+$ST2$^+$ thymic-derived Treg cells and suppresses intestinal inflammation

Ding et al.

- Supplementary Figures and Supplementary Figure legends
- Source Data of Supplementary Figures

Supplementary Fig.1

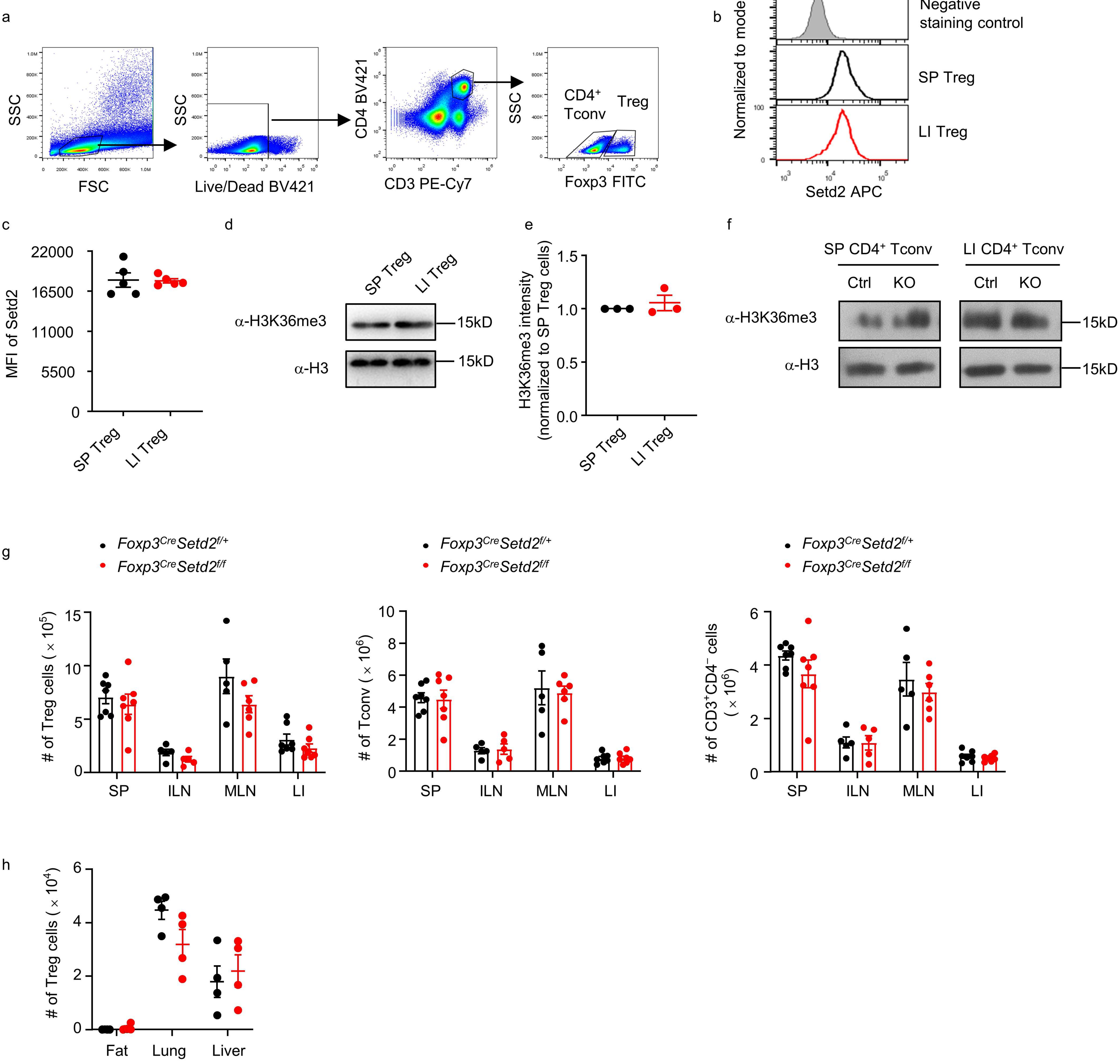

Supplementary Figure 1 Expression of Setd2 and level of H3K36me3 in Treg cells

(a) Gating strategy for Treg cells and Tconv cells (T conventional cells, $CD3^{+}CD4^{+}Foxp3^{-}$ cells) for analyzing Setd2 expression in Fig. 1a and Supplementary Fig. 1b depicted using splenocytes from *Foxp3*$^{Cre\text{-}YFP}$*Setd2*$^{f/+}$ mice. (b-c) Expression of Setd2 in splenic (SP) or large intestinal (LI) Treg cells ($CD3^{+}CD4^{+}Foxp3^{+}$) from wild-type (WT) mice was analyzed by flow cytometry (n=5 per group). (b) Histogram of Setd2 expression was shown. Secondary antibody only on splenocytes from WT mice was used as a negative staining control. (c) Mean fluorescence intensity (MFI) of Setd2 gated on Treg cells was shown. (d and e) SP or LI Treg cells ($CD3^{+}CD4^{+}$Foxp3-YFP^{+}) were purified from *Foxp3*$^{Cre\text{-}YFP}$ mice. Level of H3K36me3 and H3 was analyzed by western blot (n=3 per group). (e) Intensity of H3K36me3

signal normalized to SP Treg cells was analyzed by ImageJ. (f) LI and SP $CD4^+$ Tconv cells ($CD3^+CD4^+$Foxp3-YFP^-) were purified from littermate *Foxp3*$^{Cre\text{-}YFP}$*Setd2*$^{f/+}$ (Ctrl) or *Foxp3*$^{Cre\text{-}YFP}$*Setd2*$^{f/f}$ (KO) mice and level of H3K36me3 and H3 was analyzed by western blot. (g and h) Absolute numbers of Treg cells, Tconv cells and $CD3^+CD4^-$ cells from different organs. Fat tissues are epididymal adipose tissues. ILN, inguinal lymph nodes; MLN, mesenteric lymph nodes. (SP and LI: n=7 per group; ILN: n=5 per group; MLN: n=5 for Ctrl and n=6 for KO; Fat, lung and liver: n=4 per group). (c, e, g and h) Data are means ± SEM. (a-c, g and h) Representative of 2-3 independent experiments. (e) Data were pooled from 3 mice for each genotype. Source data are provided as a Source Data file.

Supplementary Fig.2

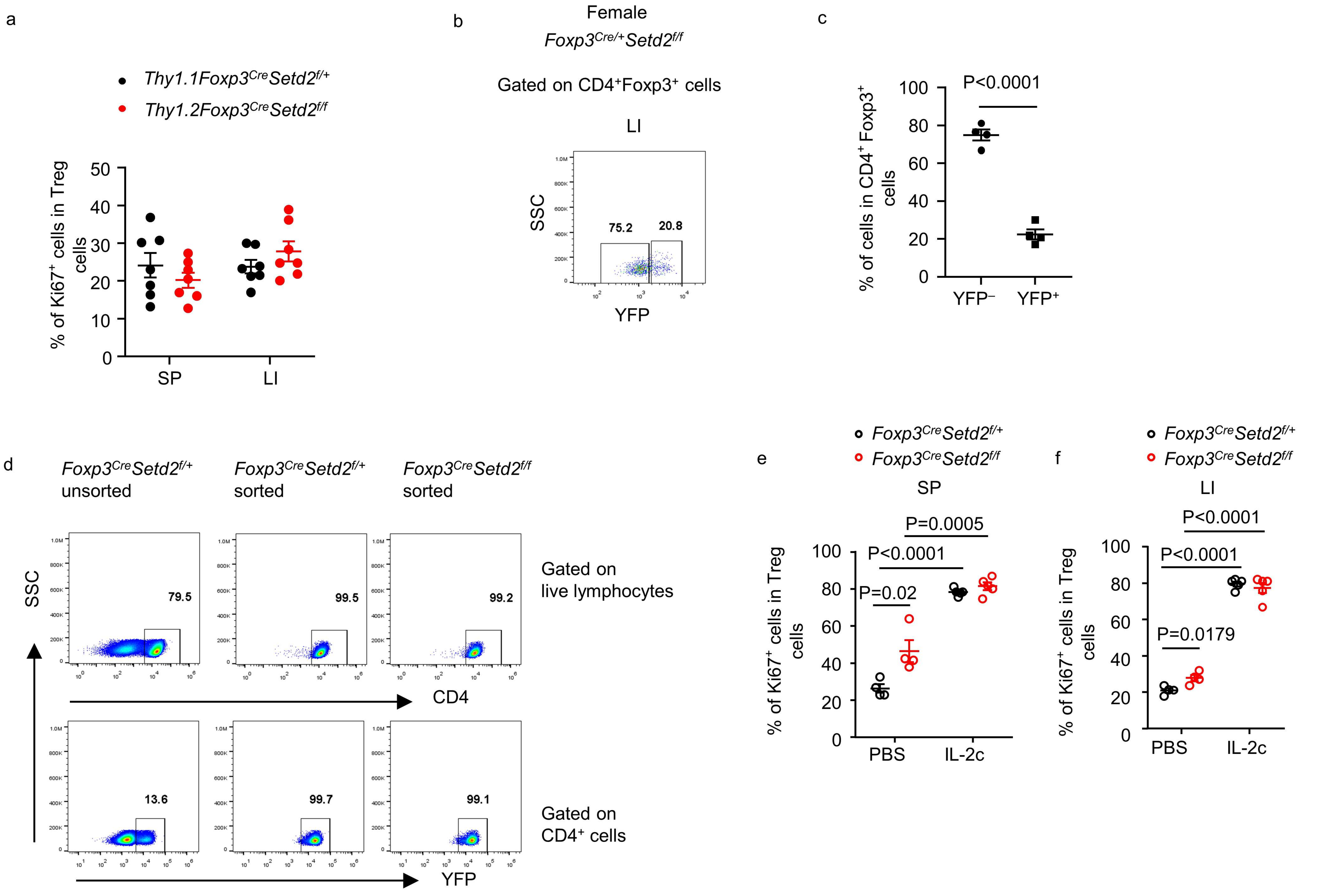

Supplementary Figure 2 Setd2-deficient Treg cells have no defect in cell proliferation

(a) Mixed bone marrow chimeric mouse was generated as described in Figure 2a. Recipient mice were sacrificed for analysis 6-8 weeks after transfer. Percentage of Ki67$^+$ cells in Treg cells (CD3$^+$CD4$^+$Foxp3$^+$) from different donor origins was analyzed by flow cytometry (n=7 per group). (b and c) Large intestinal lamina propria lymphocytes were isolated from female *Foxp3$^{Cre/+}$Setd2$^{f/f}$* mice (n=4). (b) Expression of YFP gated on gated on CD4$^+$Foxp3$^+$ cells were analyzed by flow cytometry. (c) Percentages of YFP$^+$ or YFP$^–$ cells in CD4$^+$Foxp3$^+$ cells are shown. (d) Sorting purity for cells used for adoptive transfer in Fig. 2d-2g. Cells were enriched with Dynabeads Untouched Mouse CD4 Cells kit before sorted using flow cytometry. (e and f) Mice of indicated genotypes were treated with PBS or IL-2c. Percentages of Ki67$^+$ cells in Treg cells were analyzed by flow cytometry (PBS n=4 per group; IL-2c n=5 per group). (a, b and e-f) SP, spleen; LI, large intestine. (a-c and e-f) Representative of 2-3 independent experiments. (a, c and e-f) Data are means ± SEM. Source data are provided as a Source Data file.

Supplementary Fig.3

a

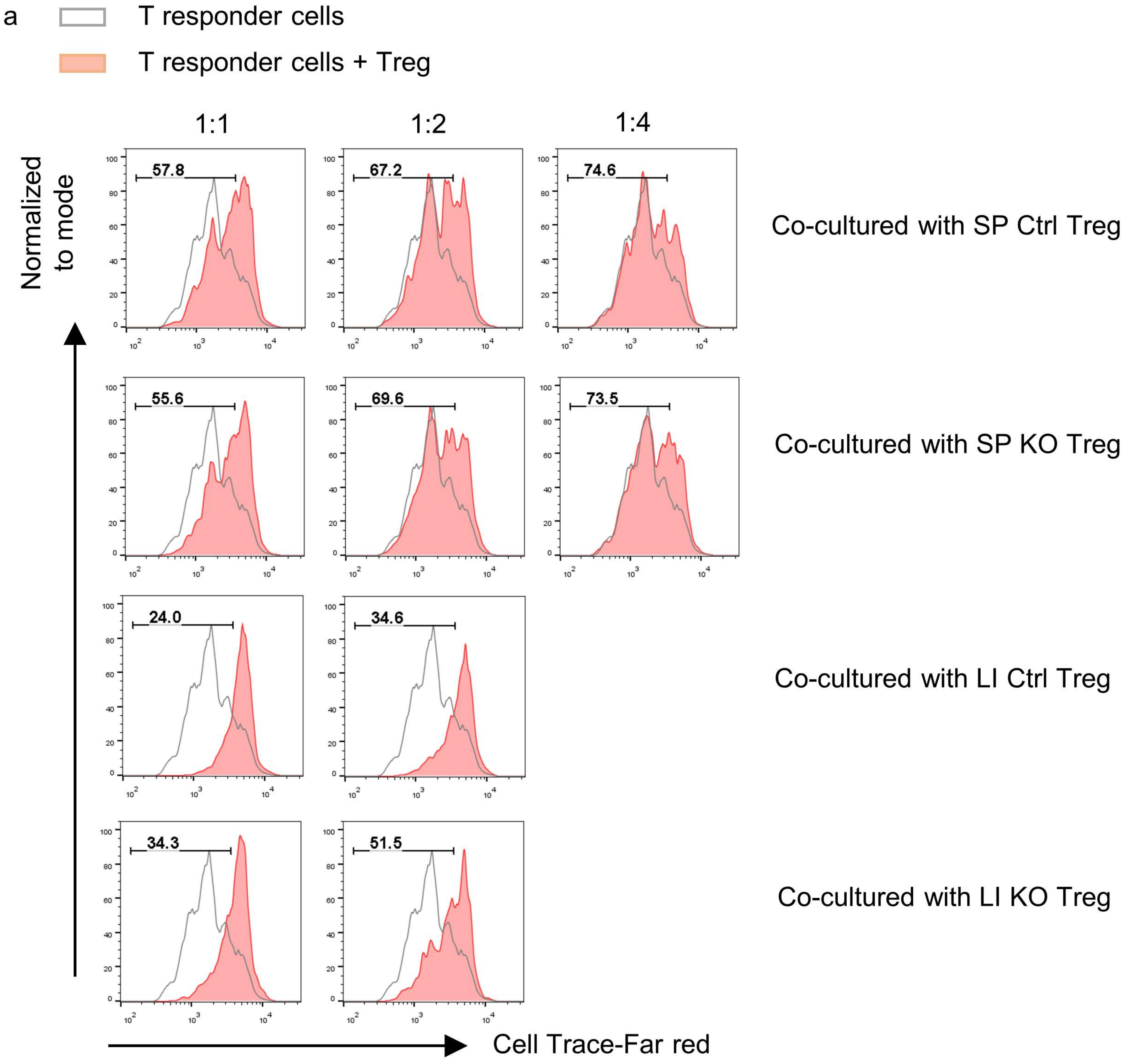

b

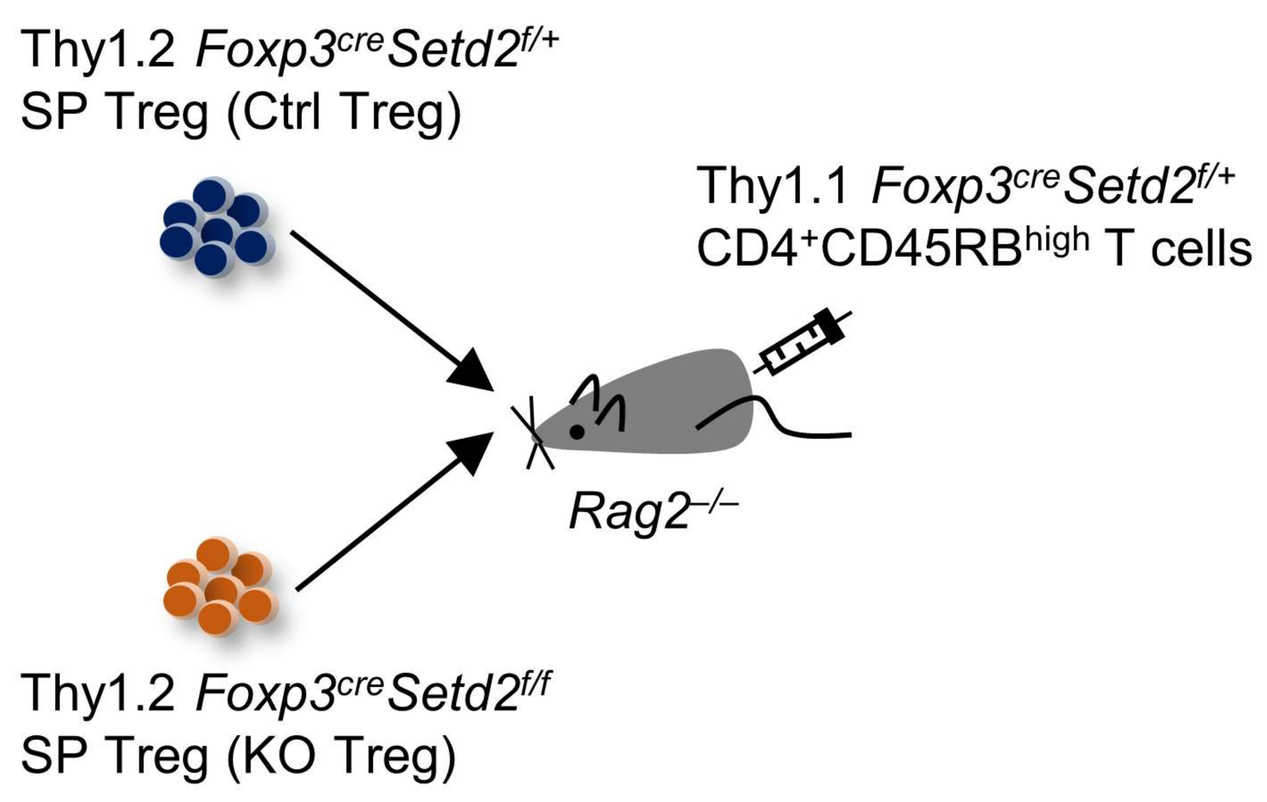

c

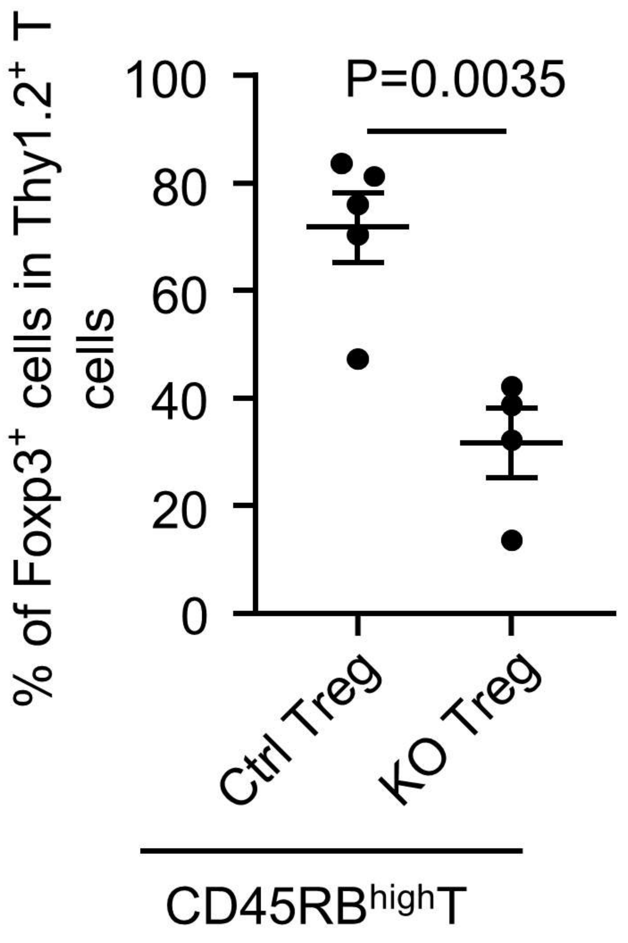

d

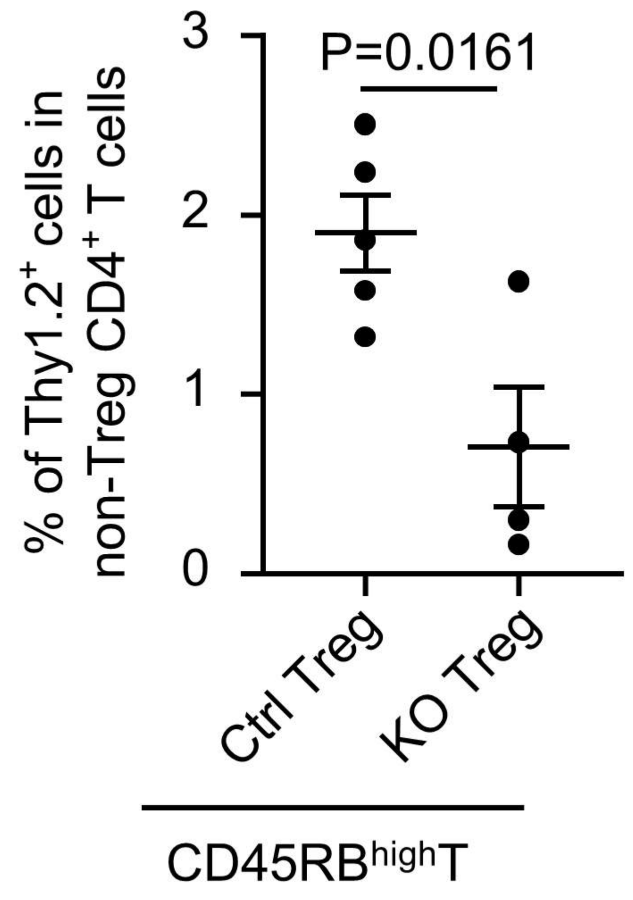

e

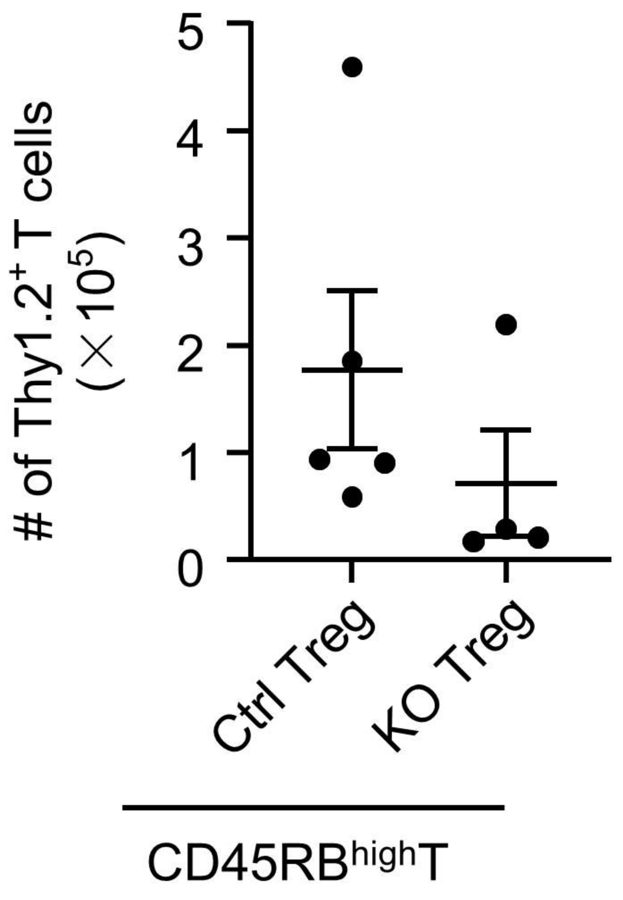

Supplementary Figure 3 Suppressive function of Setd2-deficient splenic and intestinal Treg cells

(a) T responder cells ($CD4^+CD62L^{high}CD44^{low}YFP^–$) cells were sorted from Thy1.1 *Foxp3*$^{Cre\text{-}YFP}$ mice and labeled with Cell Trace Far red. Treg cells ($CD3^+CD4^+$Foxp3-YFP^+) were sorted from spleen (SP) or large intestine (LI) of Thy1.2 *Foxp3*$^{Cre\text{-}YFP}$*Setd2*$^{f/+}$ or *Foxp3*$^{Cre\text{-}YFP}$*Setd2*$^{f/f}$ mice. Tconv cells of equal numbers were cultured with Treg cells at indicated ratios (Treg: Tconv). Expression of Cell Trace Far red gated on $CD4^+Thy1.1^+$ cells was analyzed on day 3. The percentages of proliferative cells were analyzed based on the Cell Trace Far red staining indicating the non-divided T responder cells. Data are representative of 2 independent experiments. (b-e) $Thy1.1^+$ $CD45RB^{high}$T cells ($CD3^+CD4^+$Foxp3-$YFP^–CD45RB^{high}$) with or without $Thy1.2^+$ splenic Treg cells ($CD3^+CD4^+$Foxp3-YFP^+) purified from mice of indicated genotypes were transferred to *Rag2*$^{–/–}$ mice which were sacrificed for analysis 12-14 weeks later. Large intestinal lamina propria lymphocytes were isolated from recipient mice and analyzed by flow cytometry (Ctrl n=5, KO n=4). Percentages of $Foxp3^+$ cells in $Thy1.2^+$ T ($CD4^+TCR\beta^+Thy1.2^+$) cells (c), percentages of $Thy1.2^+$ cells in non-Treg $CD4^+$ T cells ($CD4^+$ $TCR\beta^+Foxp3^–$ cells) and absolute numbers of $Thy1.2^+$ T cells (d) are shown. (c-e) Data are means $\pm$ SEM. Representative of 2 independent experiments. Source data are provided as a Source Data file.

Supplementary Fig.4

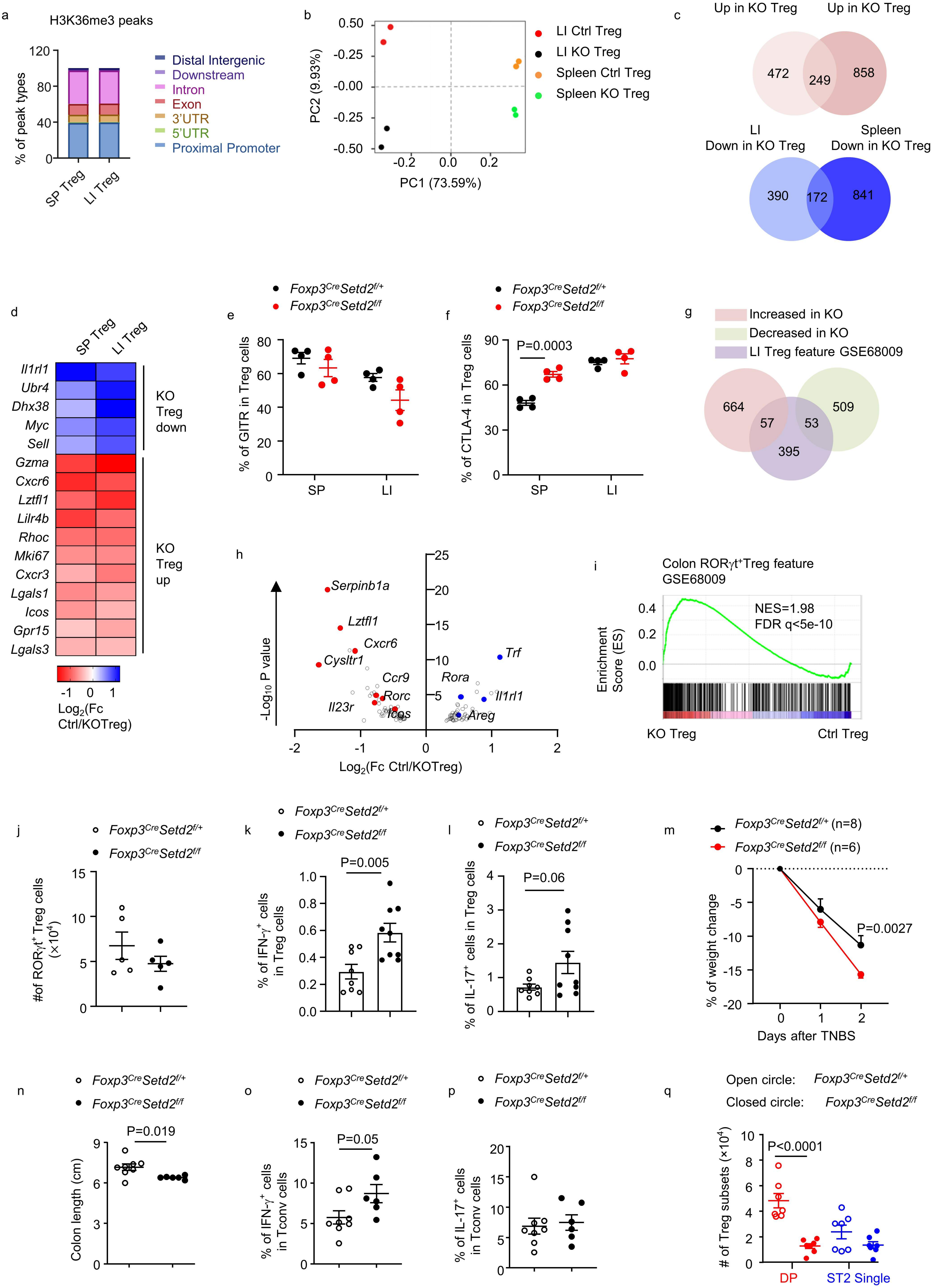

Supplementary Figure 4 Setd2 is required for Treg cells to suppress Th1 responses in TNBS colitis

(a) Proportions of different peaks from H3K36me3 ChIP-seq analysis on Treg cells ($CD3^+CD4^+$Foxp3-YFP^+) sorted from spleen (SP) or large intestine (LI) of $Foxp3^{Cre\text{-}YFP}$ mice. (b-d and g-i) SP and LI Treg cells sorted from $Foxp3^{Cre\text{-}YFP}Setd2^{f/+}$ (Ctrl) or $Foxp3^{Cre\text{-}YFP}Setd2^{f/f}$ (KO) mice were subjected to RNA-seq analysis. (b) PCA analysis based on z score of gene FPKM values. (c) Venn gram showing numbers of overlapped up or down-regulated genes in KO Treg cells. (d) Heatmap shows list of commonly changed genes in KO splenic and LI Treg cells. (e and f) LI lamina propria lymphocytes (LPLs) were isolated (n=4 per group). Percentages of $GITR^+$ (e) and CTLA-4^+ (f) cells in SP or LI Treg cells were analyzed by flow cytometry. (g)Venn gram showed genes with significantly changed expression in KO Treg cells of the LI overlapped with colonic Treg signature genes. (h) Volcano plot for the overlapped genes in (g) based on Log_2(Fold change Ctrl/KO LI Treg) and $-Log_{10}$ (p value) of intestinal Treg RNA-seq analysis. (i) GSEA analysis based on $ROR\gamma t^+$ Treg feature. (j-l) LPLs were isolated from the LI. (j) Absolute numbers of $ROR\gamma t^+$ Treg cells ($Helios^-ROR\gamma t^+$) (n=5 per group). (k and l) Cells were stimulated with PMA and ionomycin (Ctrl n=8, KO n=9). Percentages of IFN-γ^+ cells (k) and IL-17^+ cells (l) in Treg cells were analyzed by flow cytometry. (m-p) Mice were induced with TNBS colitis and sacrificed for analysis on day 2 (Ctrl n=8, KO n=6). (m) Percentage of weight change. (n) Lengths of colons. (o and p) LI LPLs were stimulated with PMA and ionomycin. Flow cytometry analysis on percentages of IFN-γ^+ cells (o) and IL-17^+ cells (p) in Tconv cells ($CD3^+CD4^+Foxp3^-$). (q) Absolute numbers of DP Treg cells (double positive, $Helios^+ST2^+$), ST2 single Treg cells (ST2 single positive, $Helios^-ST2^+$) (n=7 per group) from the LI. (e, f and j-q) Representative of 2-3 independent experiments. Data are means ± SEM. Source data are provided as a Source Data file.

Supplementary Fig.5

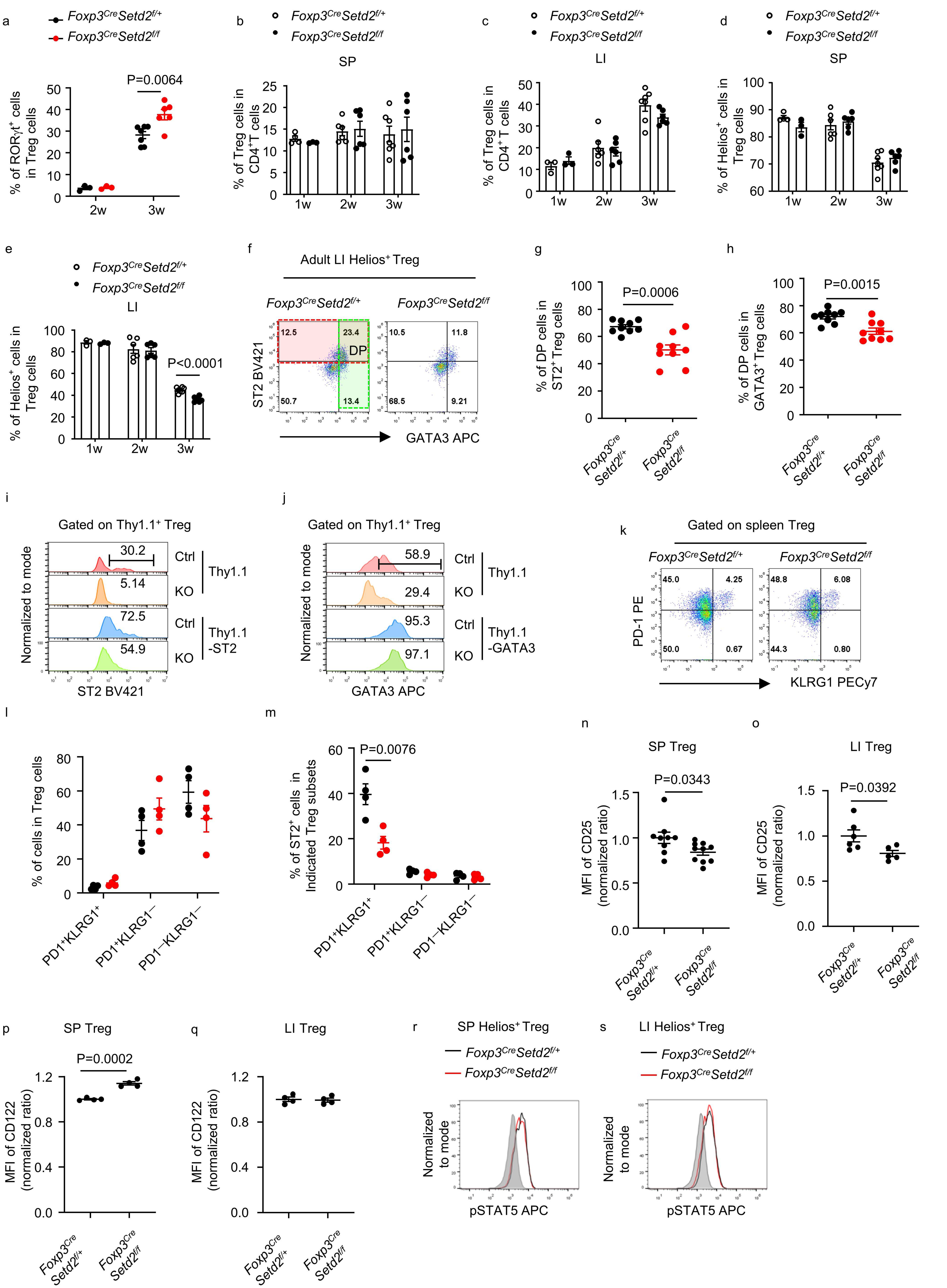

Supplementary Figure 5 Setd2 promotes the reciprocal relationship of GATA3 and ST2 in Treg cells

(a-e) Splenocytes (SP) or large intestinal (LI) LPLs were isolated from 1 week (1w), 2 weeks (2w) or 3 weeks (3w) old mice. (a) Percentage of $Helios^{-}ROR\gamma t^{+}$ cells gated on Treg cells ($CD3^{+}CD4^{+}Foxp3^{+}$) (2w: n=3 per group; 3w: Ctrl n=7, KO n=6). (b-e) Percentage of Treg cells in $CD4^{+}$ T cells and percentage of $Helios^{+}$ cells in Treg cells (1w SP: Ctrl n=4, KO n=3. 1w LI: n=3 per group; 2w SP and LI: n=6 per group; 3w SP and LI: Ctrl n=7 and KO n=6). (f-h) LI LPLs were isolated (n=9 per group). (f) Expression of GATA3 and ST2 gated on $Helios^{+}$ Treg cells. (g and h) Percentages of DP (double positive, $GATA3^{+}ST2^{+}$) cells in $ST2^{+}$ Treg cells (g) and $GATA3^{+}$ Treg cells (h). (i and j) Splenic Treg cells sorted from Ctrl ($Foxp3^{Cre\text{-}YFP}Setd2^{f/+}$) or KO ($Foxp3^{Cre\text{-}YFP}Setd2^{f/f}$) mice were cultured with α-CD3/α-CD28, IL-2 and IL-33, and were infected with retrovirus expressing Thy1.1 (i and j), Thy1.1-ST2 (i) or Thy1.1-GATA3(j). Expression of ST2 (i) and GATA3 (j) in $Thy1.1^{+}$ Treg cells ($Thy1.1^{+}Foxp3^{+}$) was analyzed. (k-m) Flow cytometry analysis of KLRG1 and PD-1 expression in splenic Treg cells from 6-12-week-old mice (n=4 per group). (l) Percentages of indicated cell subsets in Treg cells. (m) Percentages of $ST2^{+}$ cells in $PD\text{-}1^{+}KLRG1^{+}$ Treg cells, $PD\text{-}1^{+}KLRG1^{-}$ Treg cells and $PD\text{-}1^{-}KLRG1^{-}$ Treg cells. (n-q) Expression of CD25 (n and o) and CD122 (p and q) from SP (n and p) or LI (o and q) Treg cells. MFI of CD25 and CD122 were normalized to the average of $Foxp3^{Cre\text{-}YFP}Setd2^{f/+}$ group. (SP CD25 analysis: Ctrl n=9, KO n=10. LI CD25 analysis: Ctrl n=6 and KO n=5. For CD122 analysis: n=4 per group) (r and s) Splenocytes (r) or LI LPLs (s) were stimulated with recombinant murine IL-2 (50 ng/ml) for 15 min. Phosphorylated STAT5 (pSTAT5) gated on $Helios^{+}$ Treg cells was analyzed. (f-s) Representative of 2-4 independent experiments. (a-e, g-h and l-q) Data are means ± SEM. Source data are provided as a Source Data file.

Supplementary Fig.6

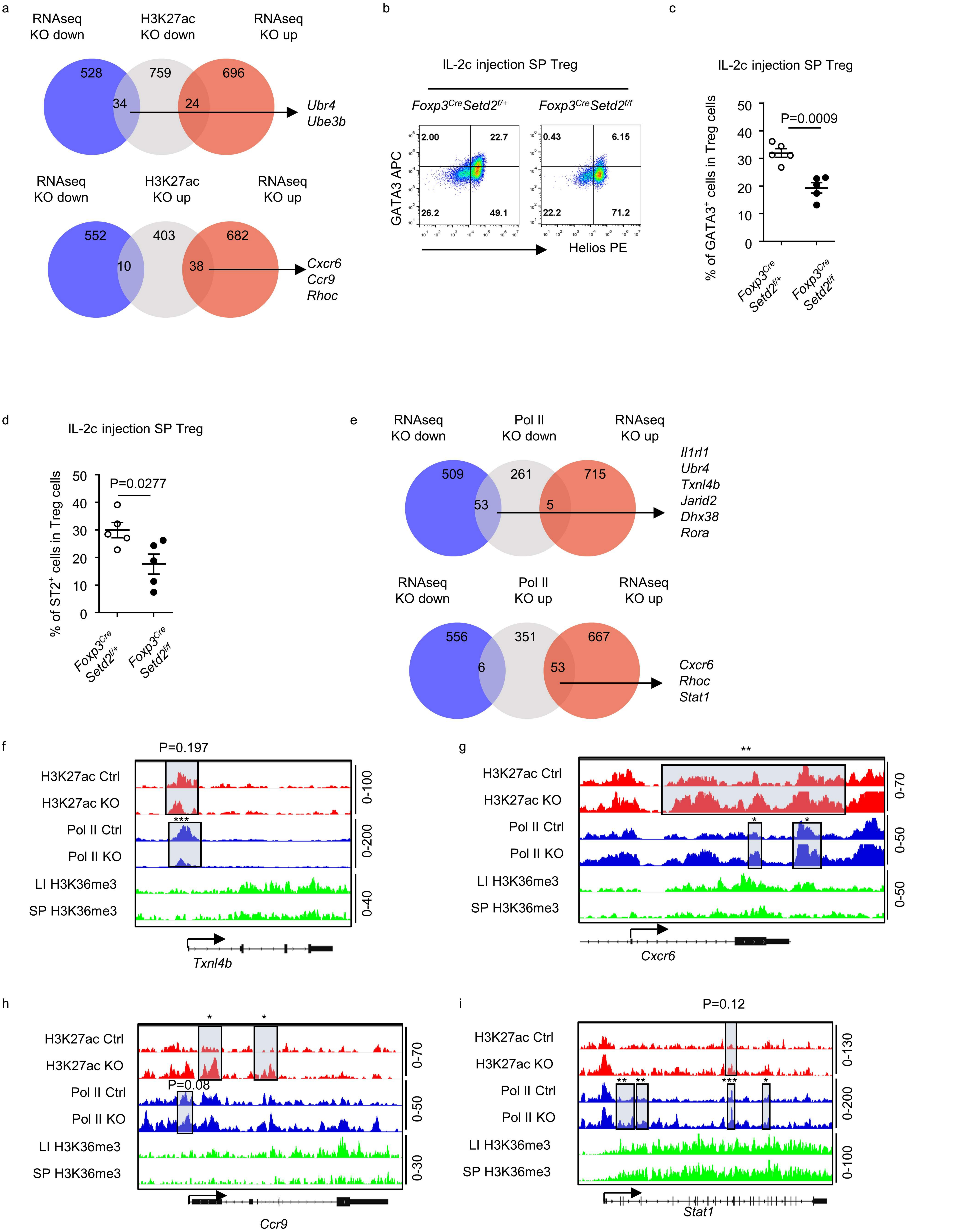

Supplementary Figure 6 Setd2 regulates gene transcription through modulation of promoter or enhancer activity in Treg cells

(a) H3K27ac CUT&Tag analysis was performed with large intestinal (LI) Treg cells purified from 2-week-old $Foxp3^{Cre\text{-}YFP}Setd2^{f/+}$ (Ctrl) or $Foxp3^{Cre\text{-}YFP}Setd2^{f/f}$ (KO) mice. RNA-seq was performed on LI Treg cells purified from adult Ctrl and KO mice. Genes with significantly decreased or increased H3K27ac peaks were overlapped with significantly decreased (down) or increased (up) expressed genes identified by RNA-seq respectively. Representative overlapped genes were shown. (b-d) Splenocytes were isolated from IL-2c-treated $Foxp3^{Cre\text{-}YFP}Setd2^{f/+}$ or $Foxp3^{Cre\text{-}YFP}Setd2^{f/f}$ mice (n=5 per group). (b) Expression of GATA3 and Helios in Treg cells ($CD4^+Foxp3^+$) was analyzed by flow cytometry. (c and d) Percentages of $GATA3^+$ cells (c) and $ST2^+$ cells (d) gated on Treg cells ($CD4^+Foxp3^+$) were shown. (e) RNA-seq was performed on LI Treg cells purified from adult Ctrl and KO mice. RNA Pol II CUT&Tag analysis was performed with splenic Treg cells nuclei of IL-2c-treated Ctrl or KO mice. Genes with significantly decreased or increased RNA Pol II peaks were overlapped with significantly decreased (down) or increased (up) expressed genes identified by RNA-seq respectively. Representative overlapped genes were shown. (f-i) Genome browser tracks of H3K27ac, RNA Pol II CUT&Tag peaks, H3K36me3 peaks at the *Txnl4b* (f), *Cxcr6* (g), *Ccr9* (h) and *Stat1*(i) locus. Boxes highlight differentially expressed peaks with or close to reach statistical significant difference analyzed by DEseq2. (c and d) Representative of 2 independent experiments. Data are means ± SEM. Source data are provided as a Source Data file.

Source Data of Supplementary Figure 1

d

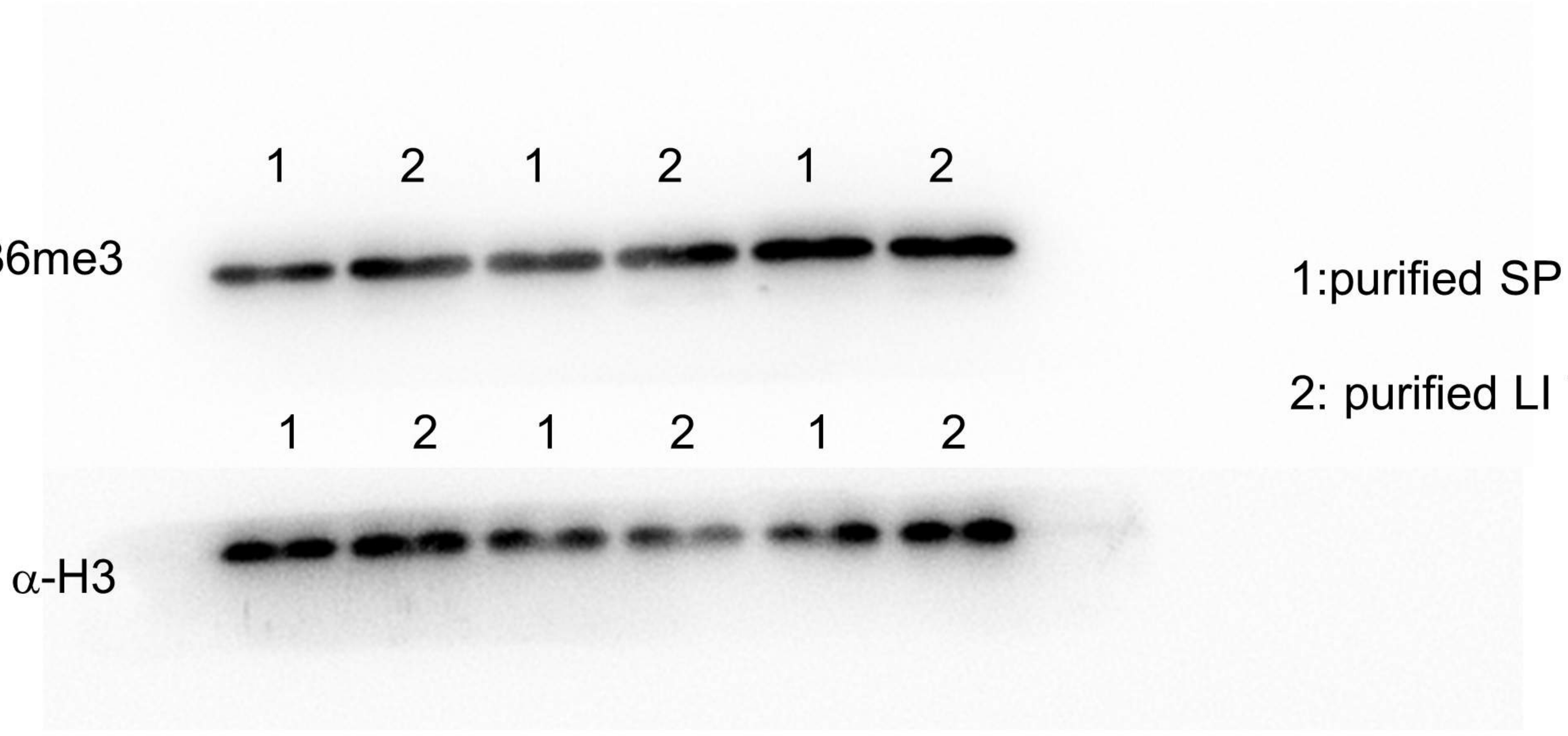

f

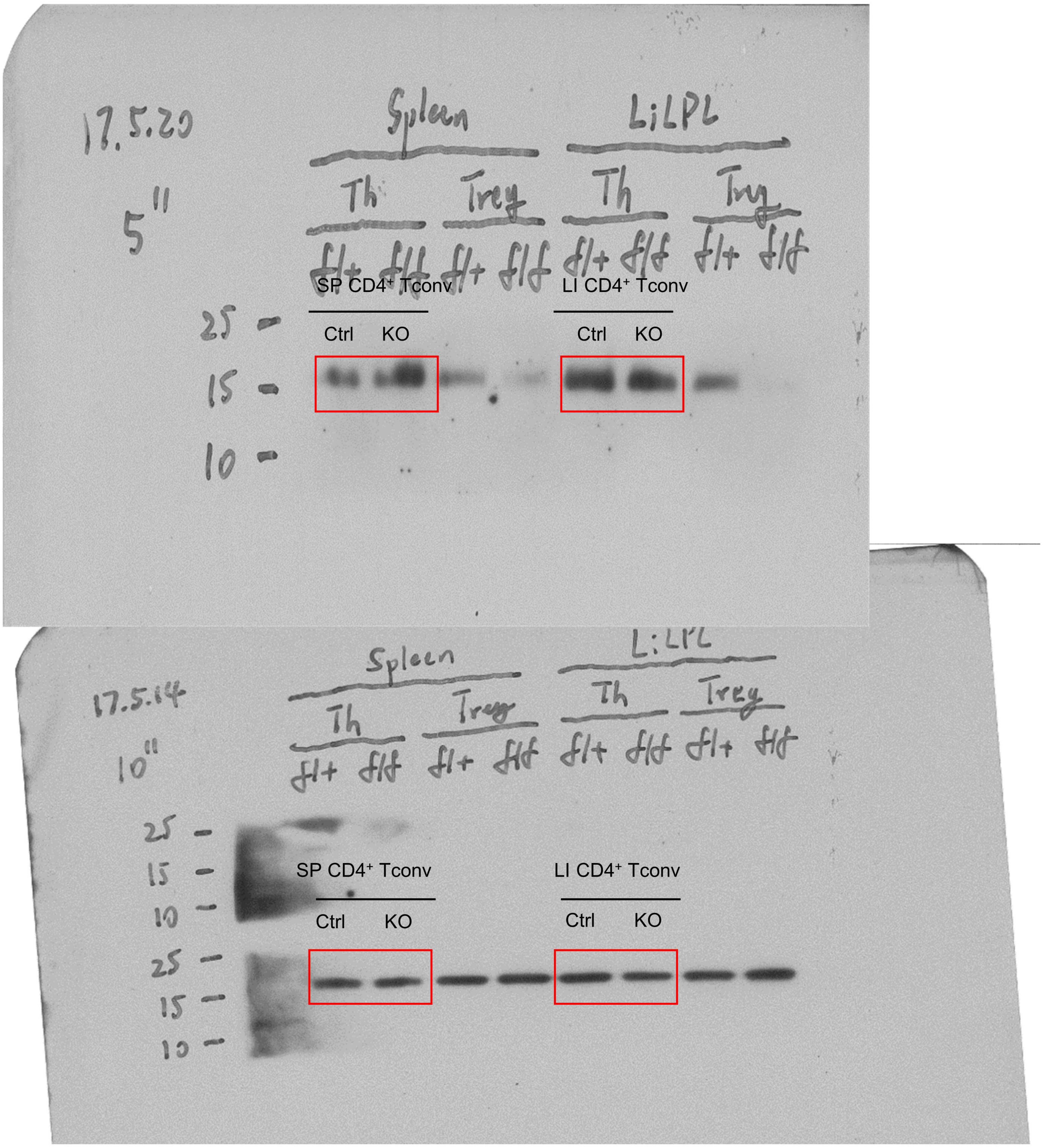

Supplement: Supplementary file 1 — Supplementary Information [file 41467_2022_35250_MOESM1_ESM.pdf]
