# Supplementary material for: Setd2 supports GATA3+ST2+ thymic-derived Treg cells and suppresses intestinal inflammation
Source: Nat Commun. 2022 Dec 3;13:7468. doi: 10.1038/s41467-022-35250-0 (PMC9719510; doi:10.1038/s41467-022-35250-0)
Supplement: Supplementary file 3 — Description of Additional Supplementary Files [file 41467_2022_35250_MOESM3_ESM.pdf]

Description of Additional Supplementary Files

Supplementary Data 1: Signature gene lists used in this study

Colonic and spleen Treg signature genes were analyzed from GSE68009. RORγt^{+} Treg signature genes ranked by fold change were analyzed from GSE68009. ST2^{+} Treg signature genes ranked by p value were analyzed from GSE136556.

Supplementary Data 2: H3K36me3 signals of colonic and spleen Treg signature genes

Log2 fold change (FC) of H3K36me3 summed signals calculated from all observed peaks annotated to each gene from SP Treg compared with LI Treg cells was shown. Colonic and spleen Treg signature genes were analyzed from GSE68009. Only genes with H3K36me3 peaks detected on both LI and SP Treg cells are shown.

Supplementary Data 3: Differentially expressed genes in Setd2-deficient Treg cells

Spleen (SP) and large intestine (LI) Treg cells sorted from *Foxp3*$^{Cre\text{-}YFP}$*Setd2*$^{f/+}$ (Ctrl) or *Foxp3*$^{Cre\text{-}YFP}$*Setd2*$^{f/f}$ (KO) mice were subjected to RNA-seq analysis. Genes differentially expressed by KOTreg were analyzed by DEseq2 and shown. Genes differentially expressed by intestinal KOTreg overlapped with colonic Treg signature genes analyzed from GSE68009 are shown.

Supplementary Data 4: Differential H3K27ac peaks in Setd2-deficient Treg cells

H3K27ac CUT&Tag analysis was performed with large intestinal (LI) Treg cells purified from 2-week-old *Foxp3*$^{Cre\text{-}YFP}$*Setd2*$^{f/+}$ (Ctrl) or *Foxp3*$^{Cre\text{-}YFP}$*Setd2*$^{f/f}$ (KO) mice. Differentially expressed H3K27ac peaks were analyzed by DEseq2.

Supplementary Data 5: Correlation of mRNA expression with H3K27ac or Pol II signals

Log2 fold change (FC) of mRNA expression, average H3K27ac signals, average Pol II signals of top significantly changed genes in mRNA expression (genes were ranked based on p value of RNA-seq regardless of upregulation or downregulation) from *Foxp3*$^{Cre\text{-}YFP}$*Setd2*$^{f/+}$ (Ctrl) compared with *Foxp3*$^{Cre\text{-}YFP}$*Setd2*$^{f/f}$ (KO) Treg cells was shown.

Supplementary Data 6: Differential Pol II peaks in Setd2-deficient Treg cells

RNA Pol II CUT&Tag analysis was performed with splenic Treg cell nuclei of IL-2c-treated *Foxp3*$^{Cre\text{-}YFP}$*Setd2*$^{f/+}$ (Ctrl) or *Foxp3*$^{Cre\text{-}YFP}$*Setd2*$^{f/f}$ (KO) mice. Differentially expressed Pol II peaks were analyzed by DEseq2.

Supplementary Data 7: Flow cytometry antibodies used in this study

Detailed information for flow cytometry antibodies used in this study are listed.
