# Supplementary material for: Setd2 supports GATA3+ST2+ thymic-derived Treg cells and suppresses intestinal inflammation
Source: Nat Commun. 2022 Dec 3;13:7468. doi: 10.1038/s41467-022-35250-0 (PMC9719510; doi:10.1038/s41467-022-35250-0)
Supplement: Supplementary file 11 — Reporting Summary [file 41467_2022_35250_MOESM11_ESM.pdf]

nature portfolio

Corresponding author(s): Li Li, Huiming Sheng and Ju Qiu

Last updated by author(s): Oct 14, 2022

Reporting Summary

Nature Portfolio wishes to improve the reproducibility of the work that we publish. This form provides structure for consistency and transparency in reporting. For further information on Nature Portfolio policies, see our Editorial Policies and the Editorial Policy Checklist.

Statistics

For all statistical analyses, confirm that the following items are present in the figure legend, table legend, main text, or Methods section.

n/a	Confirmed	
☐	☒	The exact sample size (*n*) for each experimental group/condition, given as a discrete number and unit of measurement
☐	☒	A statement on whether measurements were taken from distinct samples or whether the same sample was measured repeatedly
☐	☒	The statistical test(s) used AND whether they are one- or two-sided<br>*Only common tests should be described solely by name; describe more complex techniques in the Methods section.*
☒	☐	A description of all covariates tested
☐	☒	A description of any assumptions or corrections, such as tests of normality and adjustment for multiple comparisons
☐	☒	A full description of the statistical parameters including central tendency (e.g. means) or other basic estimates (e.g. regression coefficient) AND variation (e.g. standard deviation) or associated estimates of uncertainty (e.g. confidence intervals)
☐	☒	For null hypothesis testing, the test statistic (e.g. *F*, *t*, *r*) with confidence intervals, effect sizes, degrees of freedom and *P* value noted<br>*Give P values as exact values whenever suitable.*
☒	☐	For Bayesian analysis, information on the choice of priors and Markov chain Monte Carlo settings
☒	☐	For hierarchical and complex designs, identification of the appropriate level for tests and full reporting of outcomes
☒	☐	Estimates of effect sizes (e.g. Cohen's *d*, Pearson's *r*), indicating how they were calculated

Our web collection on statistics for biologists contains articles on many of the points above.

Software and code

Policy information about availability of computer code

Data collection	Flow cytometry data were acquired with Gallios flow cytometer (Beckman Coulter), qRT-PCR data were acquired with QuantStudio6 Q-PCR System (Angilent), RNA sequencing data were acquired with Illumina HiSeqTM4000, CHIP-seq data were acquired with Illumina NextSeq 500, CUT&Tag data were acquired with Illumina NovaSeq platform, histologic images were acquired using Olympus system, WB images were acquired using the Tanon-5200Multi system , ELISA data were acquired with a spectrophotometer (BioTek).
Data analysis	Flow cytometry data were analyzed with FlowJo Version 10.<br>Quantification of WB signal strength was performed with ImageJ.<br>Statistical analyses were performed by GraphPad Prism 8.0.<br>The raw reads of LI RNA-seq were mapped to Mouse Genome Assembly GRCm38 by STAR v2.5. Gene expression quantification was called by RSEM v1.2 with default parameters on GENCODE mouse M16 gene annotation file. Differential expression analysis was performed by Bioconductor package edgeR v3.18.1.<br>SP Tregs RNA-seq analysis was performed using the Galaxy web platform public server (https://usegalaxy.org). Quality filtered reads were mapped to mouse genome (mm10) using HISAT2(Galaxy Version 2.2.1). Gene expression quantification was called by featureCounts (Galaxy Version 2.0.1) and annotated by annotateMyIDs (Galaxy Version 3.12.0).. Differential expression analysis was performed by edgeR (Galaxy Version 3.34.0).<br>CUT&Tag raw data were evaluated with FastQC (Version 0.11.9). CUT&Tag Data analysis was performed using the Galaxy web platform public server (https://usegalaxy.org). Quality filtered reads were mapped to mouse genome (mm10) using Bowtie2 (Galaxy Version 2.3.4.3+galaxy0), and only uniquely mapped reads were kept. Duplicates reads were removed with MarkDuplicates (Galaxy Version 2.18.2.2). Peak calling was done using MACS2 (Galaxy Version 2.1.1.20160309.6) with p-value of 0.01 as cutoff against IgG control. Identified peaks were merged with “bedtools Multiple Intersect”(Galaxy Version 2.29.2) followed by “bedtools MergeBED” (Galaxy Version 2.29.2) function. Peak annotation was performed with “ChIPseeker”(Galaxy Version 1.18.0+galaxy1) .Bigwig files were generated using Bamcoverage (Galaxy Version 3.3.2.0.0) function normalized to reads per kilobase per millon (RPKM). Raw counts based on BED file of merged peaks were extracted using bedtools MultiCovBed (Galaxy Version 2.29.2). Differentially expressed peaks were analyzed using DESeq2 (Galaxy Version 2.11.40.6+galaxy1).

Visualization of read count data was performed by converting raw bam files to bigwig files using IGV tools (Version 2.4.13).
Gene set enrichment analysis (GSEA) was performed using GSEA software (Version 4.1.0, Broad Institute).

For manuscripts utilizing custom algorithms or software that are central to the research but not yet described in published literature, software must be made available to editors and reviewers. We strongly encourage code deposition in a community repository (e.g. GitHub). See the Nature Portfolio guidelines for submitting code & software for further information.

Data

Policy information about availability of data

All manuscripts must include a data availability statement. This statement should provide the following information, where applicable:
- Accession codes, unique identifiers, or web links for publicly available datasets
- A description of any restrictions on data availability
- For clinical datasets or third party data, please ensure that the statement adheres to our policy

In this research: The Treg H3K36me3 ChIP-seq data, Treg RNA-seq data, Treg H3K27ac CUT&Tag and Treg RNA Pol II CUT&Tag data generated in this study have been deposited in National Omics Data Encyclopedia (NODE) database under accession code OEP002600[https://www.biosino.org/node/project/detail/OEP002600] and the
Gene Expression Omnibus public database (GEO) database under accession code GSE182845[https://www.ncbi.nlm.nih.gov/geo/query/acc.cgi?acc=GSE182845]. Spleen Treg RNA-seq data generated in this study have also been deposited in NODE database under accession code OEP003364 [https://www.biosino.org/node/project/detail/OEP003364]. Published dataset used by this research: Spleen and colonic signature genes and RORgt+ Treg signature genes were analyzed from GSE68009 [https://www.ncbi.nlm.nih.gov/geo/query/acc.cgi?acc=GSE68009]68; ST2+ Treg signature genes were analyzed from GSE136556 [https://www.ncbi.nlm.nih.gov/geo/query/acc.cgi?acc=GSE136556]44; Treg H3K4me1 ChIP-seq data was analyzed from NCBI SRA database with SRA accession number is DRP003376 [https://trace.ncbi.nlm.nih.gov/Traces/index.html?view=study&acc=DRP003376]69. Source data are provided with this paper. Information required for reanalyzing data from this paper is available from the corresponding author upon reasonable request.

Field-specific reporting

Please select the one below that is the best fit for your research. If you are not sure, read the appropriate sections before making your selection.

[x] Life sciences [ ] Behavioural & social sciences [ ] Ecological, evolutionary & environmental sciences

For a reference copy of the document with all sections, see nature.com/documents/nr-reporting-summary-flat.pdf

Life sciences study design

All studies must disclose on these points even when the disclosure is negative.

Sample size	The number of experiment is not less than two or the sample size is not less than 3 to meet the least requirements for statistical analyses.
Data exclusions	Flow cytometry data for some samples were excluded from the statisical analysis when there was technical issues for flow cytometry staining, or when the cell events used for analysis were sharply less (8 fold less) compared with samples from the same batch of experiments, which were considered as technical problems.
Replication	Both in vitro and in vivo experiments were performed with at least 3 biological replicated or at least two independent repeats.
Randomization	Littermate mice were randomly grouped into control and treatment groups for all experiments in this study. The human sample was collected randomly. Experimental units including mouse cages, culture wells were randomly organized in this study. Orders or treatments in regard to cage location for mouse experiments, and well location for cell culture experiments were random to avoid confounders.
Blinding	For quantifications that were done with histological sections, blinding was performed by labeling the sections numerically without prior knowledge of the genotype/treatment of the sample. The other samples and analyses were not blinded to the authors. All other measurements and analyses were carried out by the authors, who need to know the cage location, well location, and sample information at all stages of experiments. In addition, gating strategies for flow cytometry analysis were kept the same for control and experimental groups. The above issues are unlikely to cause any bias for data analysis, and blinding are therefore not relevant to these experiments.

Behavioural & social sciences study design

All studies must disclose on these points even when the disclosure is negative.

Study description	*Briefly describe the study type including whether data are quantitative, qualitative, or mixed-methods (e.g. qualitative cross-sectional, quantitative experimental, mixed-methods case study).*
Research sample	*State the research sample (e.g. Harvard university undergraduates, villagers in rural India) and provide relevant demographic information (e.g. age, sex) and indicate whether the sample is representative. Provide a rationale for the study sample chosen. For studies involving existing datasets, please describe the dataset and source.*

Sampling strategy	*Describe the sampling procedure (e.g. random, snowball, stratified, convenience). Describe the statistical methods that were used to predetermine sample size OR if no sample-size calculation was performed, describe how sample sizes were chosen and provide a rationale for why these sample sizes are sufficient. For qualitative data, please indicate whether data saturation was considered, and what criteria were used to decide that no further sampling was needed.*
Data collection	*Provide details about the data collection procedure, including the instruments or devices used to record the data (e.g. pen and paper, computer, eye tracker, video or audio equipment) whether anyone was present besides the participant(s) and the researcher, and whether the researcher was blind to experimental condition and/or the study hypothesis during data collection.*
Timing	*Indicate the start and stop dates of data collection. If there is a gap between collection periods, state the dates for each sample cohort.*
Data exclusions	*If no data were excluded from the analyses, state so OR if data were excluded, provide the exact number of exclusions and the rationale behind them, indicating whether exclusion criteria were pre-established.*
Non-participation	*State how many participants dropped out/declined participation and the reason(s) given OR provide response rate OR state that no participants dropped out/declined participation.*
Randomization	*If participants were not allocated into experimental groups, state so OR describe how participants were allocated to groups, and if allocation was not random, describe how covariates were controlled.*

Ecological, evolutionary & environmental sciences study design

All studies must disclose on these points even when the disclosure is negative.

Study description	*Briefly describe the study. For quantitative data include treatment factors and interactions, design structure (e.g. factorial, nested, hierarchical), nature and number of experimental units and replicates.*
Research sample	*Describe the research sample (e.g. a group of tagged Passer domesticus, all Stenocereus thurberi within Organ Pipe Cactus National Monument), and provide a rationale for the sample choice. When relevant, describe the organism taxa, source, sex, age range and any manipulations. State what population the sample is meant to represent when applicable. For studies involving existing datasets, describe the data and its source.*
Sampling strategy	*Note the sampling procedure. Describe the statistical methods that were used to predetermine sample size OR if no sample-size calculation was performed, describe how sample sizes were chosen and provide a rationale for why these sample sizes are sufficient.*
Data collection	*Describe the data collection procedure, including who recorded the data and how.*
Timing and spatial scale	*Indicate the start and stop dates of data collection, noting the frequency and periodicity of sampling and providing a rationale for these choices. If there is a gap between collection periods, state the dates for each sample cohort. Specify the spatial scale from which the data are taken*
Data exclusions	*If no data were excluded from the analyses, state so OR if data were excluded, describe the exclusions and the rationale behind them, indicating whether exclusion criteria were pre-established.*
Reproducibility	*Describe the measures taken to verify the reproducibility of experimental findings. For each experiment, note whether any attempts to repeat the experiment failed OR state that all attempts to repeat the experiment were successful.*
Randomization	*Describe how samples/organisms/participants were allocated into groups. If allocation was not random, describe how covariates were controlled. If this is not relevant to your study, explain why.*
Blinding	*Describe the extent of blinding used during data acquisition and analysis. If blinding was not possible, describe why OR explain why blinding was not relevant to your study.*

Did the study involve field work? ☐ Yes ☒ No

Field work, collection and transport

Field conditions	*Describe the study conditions for field work, providing relevant parameters (e.g. temperature, rainfall).*
Location	*State the location of the sampling or experiment, providing relevant parameters (e.g. latitude and longitude, elevation, water depth).*
Access & import/export	*Describe the efforts you have made to access habitats and to collect and import/export your samples in a responsible manner and in compliance with local, national and international laws, noting any permits that were obtained (give the name of the issuing authority, the date of issue, and any identifying information).*
Disturbance	*Describe any disturbance caused by the study and how it was minimized.*

Reporting for specific materials, systems and methods

We require information from authors about some types of materials, experimental systems and methods used in many studies. Here, indicate whether each material, system or method listed is relevant to your study. If you are not sure if a list item applies to your research, read the appropriate section before selecting a response.

Materials & experimental systems

n/a	Involved in the study
☐	☒ Antibodies
☒	☐ Eukaryotic cell lines
☒	☐ Palaeontology and archaeology
☐	☒ Animals and other organisms
☐	☒ Human research participants
☒	☐ Clinical data
☒	☐ Dual use research of concern

Methods

n/a	Involved in the study
☐	☒ ChIP-seq
☐	☒ Flow cytometry
☒	☐ MRI-based neuroimaging

Antibodies

Antibodies used

Mouse flow cytometry antibodies:
Affinity Purified anti-mouse CD16/32 - blocks Fc binding Thermo Fisher Scientific 14-0161-85 1:100
LIVE/DEAD™ Fixable Violet Dead Cell Stain Kit L34955 1:1000
LIVE/DEAD™ Fixable Violet Dead Cell Stain Kit L10120 1:1000
Zombie Yellow™ Fixable Viability Kit Biolegend 423103 1:500
PerCP-Cyanine5.5 anti-mouse CD11b (M1/70) Thermo Fisher Scientific 45-0112-82 1:400
pecy7 anti-mouse CD11c (N418) Thermo Fisher Scientific 25-0114-82 1:400
PE anti-mouse CD25(PC61.5) Thermo Fisher Scientific 12-0251-82 1:100
PE anti-mouse CD279(PD-1) (J43) Thermo Fisher Scientific 12-9985-81 1:50
PerCP-Cyanine5.5 anti-mouse CD3e (145-2C11) Thermo Fisher Scientific 45-0031-80 1:400
APC anti-mouse CD3e (145-2C11) Thermo Fisher Scientific 17-0031-63 1:400
pecy7 anti-mouse CD4 (GK1.5) Thermo Fisher Scientific 25-0041-82 1:400
APC-eFluor® 780 anti-mouse CD4 (GK1.5) Thermo Fisher Scientific 47-0041-82 1:400
eFluor™ 450 anti-mouse CD4 (GK1.5) Thermo Fisher Scientific 48-0041-82 1:400
pecy7 anti-mouse CD8 (53-6.7) Thermo Fisher Scientific 25-0081-81 1:400
pecy7 anti-mouse/human CD44 (IM7) Thermo Fisher Scientific 25-0441-81 1:200
PE anti-mouse CD62L (MEL-14) Thermo Fisher Scientific 12-0621-82 1:200
PE anti-mouse CD45RB (C363.16A) Thermo Fisher Scientific 12-0455-82 1:200
PE anti-mouse/Rat CD90.1 (HIS51) Thermo Fisher Scientific 12-0900-81 1:200
pecy7 anti-mouse CD90.2 (30-H12) Biolegend 105325 1:200
BV421 anti-mouse CD122 (TM-BETA 1) BD Biosciences 562960 1:100
anti- Cleaved Caspase3 (5A1E) Cell Signaling Technology 9664S 1:400
APC anti-mouse CTLA-4(CD152) (UC10-4B9) Thermo Fisher Scientific 17-1522-82 1:200
FITC anti-mouse/Rat Foxp3 (FJK-16s) Thermo Fisher Scientific 11-5773-82 1:200
APC anti-mouse/Rat Foxp3 (FJK-16s) Thermo Fisher Scientific 17-5773-82 1:100
pecy7 anti-mouse GATA3 (L50-823) BD Biosciences 560405 1:20
APC anti-mouse GATA3 (L50-823) BD Biosciences 560068 1:50
APC anti-mouse GITR(CD357) (DTA-1) Thermo Fisher Scientific 17-5874-81 1:200
PE anti-mouse/human Helios (22F6) Biolegend 137216 1:50
pecy7 anti-mouse/human Helios (22F6) Biolegend 137235 1:20
pecy7 anti-mouse IFN-γ (XMG1.2) Thermo Fisher Scientific 25-7311-82 1:200
pecy7 anti-mouse IL-13 (eBio13A) Thermo Fisher Scientific 25-7133-80 1:200
PerCP-Cyanine5.5 anti-mouse/Rat IL-17a (eBio17B7) Thermo Fisher Scientific 45-7177-80 1:200
PE anti-mouse IL-4 (11B11) Thermo Fisher Scientific 12-7041-81 1:100
PE anti-mouse/human IL-5 (TRFK5) Thermo Fisher Scientific 12-7052-81 1:200
pecy7 anti-mouse/Rat Ki67 (SolA15) Thermo Fisher Scientific 25-5698-80 1:200
pecy7 anti-mouse Klrg1 (2F1) Thermo Fisher Scientific 25-5893-80 1:200
APC anti-mouse Ly-6G (1A8-Ly6g) Thermo Fisher Scientific 17-9668-80 1:200
PE anti-mouse RORγt (B2D) Thermo Fisher Scientific 12-6981-82 1:200
APC anti-mouse RORγt (B2D) Thermo Fisher Scientific 17-6981-80 1:200
PE anti-mouse/human RORγt (AFKJS-9) Thermo Fisher Scientific 12-6988-82 1:200
anti-mouse/human Setd2 (E4W8Q) Cell Signaling Technology 80290S 1:400
PE anti-mouse Siglec-F (E50-2440) BD Biosciences 552126 1:200
BV421 anti-mouse ST2 (U29-93) BD Biosciences 566309 1:200
PE anti-mouse ST2 (DJ8) MP biomedicals 101001PE 1:800

Human flow cytometry antibodies:
PE anti-human CD127 (A019D5) Biolegend 351303 1:40
PerCP-Cyanine5.5 anti-human CD127 (A019D5) Biolegend 351321 1:100
pecy7 anti-human CD25 (BC96) Biolegend 302611 1:40
FITC anti-human CD3e (UCHT1) Biolegend 300406 1:20
FITC anti-human CD4 (OKT-4) Thermo Fisher Scientific 11-0048-42 1:40
BV421 anti-human CD4 (RPA-T4) BD Biosciences 562425 1:100
Alexa Fluor® 647 anti-mouse/human/rat Foxp3 (150D) Biolegend 320013 1:100
pecy7 anti-human GATA3 (TWAJ) Thermo Fisher Scientific 25-9966-41 1:20
PerCP-Cyanine5.5 anti-human IL-13 (JES10-5A2) Biolegend 501912 1:20

Flow cytometry second antibodies:
Alexa Fluor 488 Donkey anti-Rabbit IgG (H+L) Thermo Fisher Scientific R37118 1:250
PE Goat Anti-Rabbit IgG (H+L) Jackson ImmunoResearch 111-116-144 1:200
Alexa Fluor 647 Goat Anti-Rabbit IgG (H+L) Jackson ImmunoResearch 111-605-003 1:200

WB antibodies:Anti-H3K36me3
antibody Abcam ab9050 1:1000Histone
H3 antibody Cell Signaling Technology 9715S 1:1000
HRP-conjugated Goat Anti-Rabbit IgG (H+L) Proteintech SA00001-2 1:1000

CUT&Tag antibodies:
Anti-H3K27ac antibody ABCAM ab4729 2 ug
Anti-RNA Pol II antibody(4H8) Active Motif 39497 0.5 ug
Control rabbit IgG ABCAM ab171870 2 ug
Control mouse IgG1 SouthernBiotech 0102-01 0.5 ug

Validation

LIVE/DEAD™ Fixable Violet Dead Cell Stain Kit L34955
LIVE/DEAD™ Fixable Violet Dead Cell Stain Kit L10120
Zombie Yellow™ Fixable Viability Kit Biolegend 423103
(Reactivity: Eukaryotic cells Application: FC)

PerCP-Cyanine5.5 anti-mouse CD11b (M1/70) Thermo Fisher Scientific 45-0112-82
(Reactivity: Mouse Application: FC, ICC/IF)

pecy7 anti-mouse CD11c (N418) Thermo Fisher Scientific 25-0114-82
(Reactivity: Mouse Application: FC, ICC/IF)

PE anti-mouse CD25(PC61.5) Thermo Fisher Scientific 12-0251-82 1:100
(Reactivity: Mouse Application: FC, ICC/IF)

PE anti-mouse CD279(PD-1) (J43) Thermo Fisher Scientific 12-9985-81
(Reactivity: Mouse Application: FC, ICC/IF, FN, WB, Misc)

PerCP-Cyanine5.5 anti-mouse CD3e (145-2C11) Thermo Fisher Scientific 45-0031-80
APC anti-mouse CD3e (145-2C11) Thermo Fisher Scientific 17-0031-63
(Reactivity: Mouse Application: FC, ICC/IF, FN)

pecy7 anti-mouse CD4 (GK1.5) Thermo Fisher Scientific 25-0041-82
APC-eFluor® 780 anti-mouse CD4 (GK1.5) Thermo Fisher Scientific 47-0041-82
(Reactivity: Mouse Application: FC, FN)

eFluor™ 450 anti-mouse CD4 (GK1.5) Thermo Fisher Scientific 48-0041-82
(Reactivity: Mouse Application: FC, IHC, IF, FN)

pecy7 anti-mouse CD8 (53-6.7) Thermo Fisher Scientific 25-0081-81
(Reactivity: Mouse Application: FC, FN)

pecy7 anti-mouse/human CD44 (IM7) Thermo Fisher Scientific 25-0441-81
(Reactivity: Human, Mouse Application: FC)

PE anti-mouse CD62L (MEL-14) Thermo Fisher Scientific 12-0621-82
(Reactivity: Mouse Application: FC, IV)

PE anti-mouse CD45RB (C363.16A) Thermo Fisher Scientific 12-0455-82
(Reactivity: Mouse Application: FC)

PE anti-mouse/Rat CD90.1 (HIS51) Thermo Fisher Scientific 12-0900-81
(Reactivity: Mouse, Rat Application: FC, IHC)

pecy7 anti-mouse CD90.2 (30-H12) Biolegend 105325
(Reactivity: Mouse Application: FC)

BV421 anti-mouse CD122 (TM-BETA 1) BD Biosciences 562960
(Reactivity: Mouse Application: FC)

anti-Cleaved Caspase3 (5A1E) Cell Signaling Technology 9664S
(Reactivity: Human, Mouse, Rat, Monkey Application: FC, WB, IP, IF, IHC)

APC anti-mouse CTLA-4(CD152) (UC10-4B9) Thermo Fisher Scientific 17-1522-82
(Reactivity: Mouse, Bovine, Dog, Cat, Pig, Rat Application: FC)

FITC anti-mouse/Rat Foxp3 (FJK-16s) Thermo Fisher Scientific 11-5773-82
(Reactivity: Mouse Application: FC, IHC, ICC/IF)

APC anti-mouse/Rat Foxp3 (FJK-16s) Thermo Fisher Scientific 17-5773-82
(Reactivity: Mouse Application: FC, IHC, ICC/IF, CHIP)

pecy7 anti-mouse GATA3 (L50-823) BD Biosciences 560405
APC anti-mouse GATA3 (L50-823) BD Biosciences 560068
(Reactivity: Human, Mouse Application: FC)

APC anti-mouse GITR(CD357) (DTA-1) Thermo Fisher Scientific 17-5874-81
(Reactivity: Mouse Application: FC)

PE anti-mouse/human Helios (22F6) Biolegend 137216
pecy7 anti-mouse/human Helios (22F6) Biolegend 137235
(Reactivity: Human, Mouse Application: FC)

pecy7 anti-mouse IFN-γ (XMG1.2) Thermo Fisher Scientific 25-7311-82
(Reactivity: Mouse Application: FC)

pecy7 anti-mouse IL-13 (eBio13A) Thermo Fisher Scientific 25-7133-80
(Reactivity: Mouse Application: FC)

PerCP-Cyanine5.5 anti-mouse/Rat IL-17a (eBio17B7) Thermo Fisher Scientific 45-7177-80
(Reactivity: Mouse, Rat Application: FC, IHC, FN)

PE anti-mouse IL-4 (11B11) Thermo Fisher Scientific 12-7041-81
(Reactivity: Mouse Application: FC)

PE anti-mouse/human IL-5 (TRFK5) Thermo Fisher Scientific 12-7052-81
(Reactivity: Human, Mouse Application: FC, ICC/IF)

pecy7 anti-mouse/Rat Ki67 (SolA15) Thermo Fisher Scientific 25-5698-80
(Reactivity: Human, Mouse, Dog, Cynomolgus monkey, Non-human primate, Rat Application: FC, FN)

pecy7 anti-mouse Klrg1 (2F1) Thermo Fisher Scientific 25-5893-80
(Reactivity: Mouse Application: FC)

APC anti-mouse Ly-6G (1A8-Ly6g) Thermo Fisher Scientific 17-9668-80
(Reactivity: Mouse Application: FC)

PE anti-mouse RORγt (B2D) Thermo Fisher Scientific 12-6981-82
APC anti-mouse RORγt (B2D) Thermo Fisher Scientific 17-6981-80
(Reactivity: Mouse Application: FC)

PE anti-mouse/human RORγt (AFKJS-9) Thermo Fisher Scientific 12-6988-82
(Reactivity: Human, Mouse, Rhesus monkey Application: FC, IHC)

anti-mouse/human Setd2 (E4W8Q) Cell Signaling Technology 80290S
(Reactivity: Human, Mouse, Monkey Application: WB, CHIP)

PE anti-mouse Siglec-F (E50-2440) BD Biosciences 552126
(Reactivity: Mouse Application: FC)

BV421 anti-mouse ST2 (U29-93) BD Biosciences 566309

(Reactivity: Mouse Application: FC)

PE anti-mouse ST2 (DJ8) MP biomedicals 101001PE
(Reactivity: Mouse Application: FC, IP)

Human flow cytometry antibodies:
PE anti-human CD127 (A019D5) Biolegend 351303
(Reactivity: Human, African Green, Baboon, Cynomolgus, Rhesus Application: FC)

PerCP-Cyanine5.5 anti-human CD127 (A019D5) Biolegend 351321
(Reactivity: Human, African Green, Baboon, Cynomolgus, Rhesus Application: FC)

pecy7 anti-human CD25 (BC96) Biolegend 302611
(Reactivity: Human, Baboon, Chimpanzee, Cynomolgus, Pigtailed Macaque, Rhesus Application: FC)

FITC anti-human CD3e (UCHT1) Biolegend 300406
(Reactivity: Human, Chimpanzee Application: FC)

FITC anti-human CD4 (OKT-4) Thermo Fisher Scientific 11-0048-42
(Reactivity: Human Application: FC, WB, IHC, ICC/IF)

BV421 anti-human CD4 (RPA-T4) BD Biosciences 562425
(Reactivity: Human Application: FC, IF)

Alexa Fluor® 647 anti-mouse/human/rat Foxp3 (150D) Biolegend 320013
(Reactivity: Human, Mouse, Rat, Cross-Reactivity: Cynomolgus, Rhesus, Baboon Application: FC)

pecy7 anti-human GATA3 (TWAJ) Thermo Fisher Scientific 25-9966-41
(Reactivity: Human, Mouse, Pig, Rhesus monkey Application: FC)

PerCP-Cyanine5.5 anti-human IL-13 (JES10-5A2) Biolegend 501912
(Reactivity: Human Application: FC)

Flow cytometry second antibodies:
Alexa Fluor 488 Donkey anti-Rabbit IgG (H+L) Thermo Fisher Scientific R37118
(Reactivity: Rabbit Application: FC, ICC/IF)

PE Goat Anti-Rabbit IgG (H+L) Jackson ImmunoResearch 111-116-144
(Reactivity: Rabbit Application: FC, ICC/IF)

Alexa Fluor 647 Goat Anti-Rabbit IgG (H+L) Jackson ImmunoResearch 111-605-003
(Reactivity: Rabbit Application: FC, ICC/IF)

WB antibodies:Anti-H3K36me3
antibody Abcam ab9050
(Reactivity: Human, Cow, Mouse Application: WB, CHIP, ICC/IF)Histone

H3 antibody Cell Signaling Technology 9715S
(Reactivity: Human, Mouse, Rat, Monkey, Zebrafish, Bovine, Pig Application: WB)

HRP-conjugated Goat Anti-Rabbit IgG (H+L) Proteintech SA00001-2
(Reactivity: Rabbit Application: WB, ELISA)

CUT&Tag antibodies:
Anti-H3K27ac antibody ABCAM ab4729
(Reactivity: Human, Mouse, Rat, Cow Application: ICC/IF, WB, IHC-P, ChIP, PepArr)

Anti-RNA Pol II antibody(4H8) Active Motif 39497
(Reactivity: Human, Budding Yeast, C. elegans, Mouse, Rat Application: WB, ChIP)

Control rabbit IgG ABCAM ab171870
(Application: WB, ChIP, FC)

Control mouse IgG1 SouthernBiotech 0102-01
(Application: WB, ChIP, FC, ICC/IF, ELISA, FLISA)

Eukaryotic cell lines

Policy information about cell lines

Cell line source(s)	*State the source of each cell line used.*
Authentication	*Describe the authentication procedures for each cell line used OR declare that none of the cell lines used were authenticated.*
Mycoplasma contamination	*Confirm that all cell lines tested negative for mycoplasma contamination OR describe the results of the testing for mycoplasma contamination OR declare that the cell lines were not tested for mycoplasma contamination.*
Commonly misidentified lines (See ICLAC register)	*Name any commonly misidentified cell lines used in the study and provide a rationale for their use.*

Palaeontology and Archaeology

Specimen provenance	*Provide provenance information for specimens and describe permits that were obtained for the work (including the name of the issuing authority, the date of issue, and any identifying information). Permits should encompass collection and, where applicable, export.*
Specimen deposition	*Indicate where the specimens have been deposited to permit free access by other researchers.*
Dating methods	*If new dates are provided, describe how they were obtained (e.g. collection, storage, sample pretreatment and measurement), where they were obtained (i.e. lab name), the calibration program and the protocol for quality assurance OR state that no new dates are provided.*

☐ Tick this box to confirm that the raw and calibrated dates are available in the paper or in Supplementary Information.

Ethics oversight	*Identify the organization(s) that approved or provided guidance on the study protocol, OR state that no ethical approval or guidance was required and explain why not.*

Note that full information on the approval of the study protocol must also be provided in the manuscript.

Animals and other organisms

Policy information about studies involving animals; ARRIVE guidelines recommended for reporting animal research

Laboratory animals	Rag1–/–, Rag2–/– mice, Foxp3Cre-YFP and Thy1.1 mice were purchased from Jackson laboratory. Il33–/– mouse from Riken Center for Biosystems Research (accession number CDB0631K). Setd2flox/flox(Setd2f/f) mouse was generated Dr. Li Li (Corresponding author of this paper). Mice used for in vivo studies were littermates, age matched and were 6-12 weeks old unless otherwise specified. Both male and female mice were used unless otherwise noted. All mice used in this study are on C57BL/6 background, maintained in specific pathogen-free conditions, fed with a plain commercial diet (Silaikang, Shanghai) and housed in corn-cob-bedding cages in a room with the light-dark cycle (lights on at 6:00 and off at 18:00).The mice were kept at a constant temperature of 22±3℃ and a relative humidity of 35±5%.
Wild animals	The study did not involve wild animals.
Field-collected samples	The study did not involve samples collected from field.
Ethics oversight	All animal experiments were performed in compliance with the ''Guide for the Care and Use of Laboratory Animals'' and approved by the institutional biomedical research ethics committee of the Shanghai Institutes for Nutrition and Health, Chinese Academy of Sciences. Ethics approval number:SINH-2020-QJ-2; SINH-2021-QJ-1; SINH-2022-QJ-1.

Note that full information on the approval of the study protocol must also be provided in the manuscript.

Human research participants

Policy information about studies involving human research participants

Population characteristics	Human samples were obtained from the Department of General Surgery of Tongren Hospital with informed consent.<br>Human blood samples were collected from both female and male healthy donors, with age ranging from 25 to 35 .<br><br>Flow cytometry analysis:<br>Human cancerous tissues and non-cancerous tissues (≥10 cm away from the tumor) were collected from colorectal cancer patients during surgery (Department of General Surgery of Tongren Hospital). The study was performed in strict compliance with all institutional ethical regulations.<br><br>Gender: Female: n=2 (25%), Male: n=6 (75%)<br>Age at diagnosis: <70 n=5 (62.5%), >70 n=3 (37.5%)<br>Pathological diagnosis: rectal tumor: n=6 (75%), colon tumor: n=2 (25%)

Recruitment	Cancerous cancer patients eligible for surgical reception at Department of General Surgery of Tongren Hospital were recruited to participate in an Institutional Reviews Board-approved protocol. All patients who provided informed consent had samples collected; all study procedures were conducted in strict compliance with all ethical and institutional regulations. No participant compensation was indicated in the consent form. The human samples were collected randomly, with more females than males and more rectum than colon, which we didn't think will affect the results.
Ethics oversight	Independent Ethics Committee of Shanghai Tongren Hospital. Ethics approval number:2020-043-01;2019-052-01

Note that full information on the approval of the study protocol must also be provided in the manuscript.

Clinical data

Policy information about clinical studies

All manuscripts should comply with the ICMJEguidelines for publication of clinical research and a completedCONSORT checklist must be included with all submissions.

Clinical trial registration	*Provide the trial registration number from ClinicalTrials.gov or an equivalent agency.*
Study protocol	*Note where the full trial protocol can be accessed OR if not available, explain why.*
Data collection	*Describe the settings and locales of data collection, noting the time periods of recruitment and data collection.*
Outcomes	*Describe how you pre-defined primary and secondary outcome measures and how you assessed these measures.*

Dual use research of concern

Policy information about dual use research of concern

Hazards

Could the accidental, deliberate or reckless misuse of agents or technologies generated in the work, or the application of information presented in the manuscript, pose a threat to:

No	Yes	
☒	☐	Public health
☒	☐	National security
☒	☐	Crops and/or livestock
☒	☐	Ecosystems
☒	☐	Any other significant area

Experiments of concern

Does the work involve any of these experiments of concern:

No	Yes	
☒	☐	Demonstrate how to render a vaccine ineffective
☒	☐	Confer resistance to therapeutically useful antibiotics or antiviral agents
☒	☐	Enhance the virulence of a pathogen or render a nonpathogen virulent
☒	☐	Increase transmissibility of a pathogen
☒	☐	Alter the host range of a pathogen
☒	☐	Enable evasion of diagnostic/detection modalities
☒	☐	Enable the weaponization of a biological agent or toxin
☒	☐	Any other potentially harmful combination of experiments and agents

ChIP-seq

Data deposition

☒ Confirm that both raw and final processed data have been deposited in a public database such as GEO.

☒ Confirm that you have deposited or provided access to graph files (e.g. BED files) for the called peaks.

Data access links<br>*May remain private before publication.*	https://www.biosino.org/node/project/detail/OEP002600
Files in database submission	OES111120 H3K36me3 ChIP-seq in spleen WT Treg<br>OES111121 H3K36me3 ChIP-seq in LI WT Treg

	OES111122 H3K36me3 ChIP-seq in spleen WT Treg input<br>OES111115 H3K27ac CUT&Tag in LI ctrl Treg rep1<br>OES111116 H3K27ac CUT&Tag in LI ctrl Treg rep2<br>OES111117 H3K27ac CUT&Tag in LI KO Treg rep1<br>OES111118 H3K27ac CUT&Tag in LI KO Treg rep2<br>OES111119 H3K27ac CUT&Tag in LI Treg IgG<br>OES111110 polII CUT&Tag in spleen ctrl Treg rep1<br>OES111111 polII CUT&Tag in spleen ctrl Treg rep2<br>OES111112 polII CUT&Tag in spleen KO Treg rep1<br>OES111113 polII CUT&Tag in spleen KO Treg rep2<br>OES111114 polII CUT&Tag in spleen Treg IgG
Genome browser session (e.g. UCSC)	https://genome.ucsc.edu/cgi-bin/hgTracks?db=mm10&lastVirtModeType=default&lastVirtModeExtraState=&virtModeType=default&virtMode=0&nonVirtPosition=&position=chr12%3A56694976%2D56714605&hgsid=1372567115_xWq1mTu42QVVKazdvULoJicaVaIK

Methodology

Replicates	CD3+CD4+YFP+ Tregs were pooled from7 female and 7 male mice per replicate for H3K36me3 CHIP-seq. Genomic DNA of splenic Tregs was saved as Input.<br>CD3+CD4+YFP+ Tregs were pooled from 3-5 mice per replicate for H3K27ac CUT&Tag and pooled from 2 mice per replicate for polII CUT&Tag.<br>For H3K36me3 ChIP-seq in Treg cells, 1 replicate was done.<br>For H3K27ac CUT&Tag in LI Treg cells, 2 Setd2-sufficient and 2 Setd2-deficient replicates were done, 1 IgG replicate was done.<br>For polII CUT&Tag in splenic Treg cells, 2 Setd2-sufficient and 2 Setd2-deficient replicates were done, 1 IgG replicate was done.
Sequencing depth	H3K36me3 CHIP-seq: 75 bp single end sequencing, SPTreg 36,946,521 reads; LI Treg 43,524,194 reads; Input 36,802,789 reads.<br>H3K27ac CUT&Tag, pol II CUT&Tag: 150bp paired-end sequencing, 6G raw reads.
Antibodies	Anti-Histone H3K36me3 antibody (polyclonal) Active Motif 61101 Host: Rabbit; Reactivity: Mouse, Human; Applications: ChIP, ICC, IF, WB;<br>Anti-Histone H3 (acetyl K27) antibody (polyclonal) Abcam ab4729 Host: Rabbit; Reactivity: Mouse, Rat, Cow, Human; Applications: ChIP, ICC/IF, WB, IHC-P, PepArr<br>RNA pol II antibody(Monoclonal) Active Motif 39497 Host: Mouse; Reactivity: Budding Yeast, C. elegans, Human, Mouse, Rat; Applications: ChIP, ICC, IF, WB;<br>Rabbit IgG-Isotype Control (polyclonal) Abcam ab171870 Host: Rabbit; Applications: ChIP, WB, FC<br>Mouse IgG1 (Monoclonal) SouthernBiotech 0102-01 Host: Mouse; Applications: FC, ELISA, IFC, ICC, WB
Peak calling parameters	H3K36me3 CHIP-seq: The 75-nt sequence reads are mapped to the mm10 using the BWA algorithm with default settings. Alignment information for each read is stored in the BAM format. Only reads that pass Illumina's purity filter, align with no more than 2 mismatches, and map uniquely to the genome are used in the subsequent analysis. In addition, unless stated otherwise, duplicate reads ("PCR duplicates") are removed. Then, peak calling was performed using the Galaxy web platform public server (https://usegalaxy.org) using MACS2 with threshold p-value of 0.01 as cutoff based on input.<br>H3K27ac and polII CUT&Tag: Mapping and peak calling was performed using the Galaxy web platform public server (https://usegalaxy.org). Quality filtered reads were mapped to mouse genome (mm10) using Bowtie2, and only uniquely mapped reads were kept. Duplicates reads were removed with MarkDuplicates. Peak calling was done using MACS2 with p-value of 0.01 as cutoff against IgG control.
Data quality	When performing mapping, only mapping quality higher than 20 was kept for downstream analysis, reads mapped to mitochondria genome were filtered, only properly paired reads were kept for downstream analysis. PCR duplicates were removed from analysis. All peaks are at p value <0.01; about 20% peaks are above 5-fold enrichment; 99% peaks are above 2-fold enrichment.
Software	Galaxy web platform was used for CUT&Tag analyses. The following suite have been used: BWA algorithm, Bowtie2, MarkDuplicates, MACS2, bedtools Multiple Intersect, bedtools MergeBED, ChIPseeker, Bamcoverage, bedtools MultiCovBed, DESeq2. IGV tools were used for visualization.

Flow Cytometry

Plots

Confirm that:

☒ The axis labels state the marker and fluorochrome used (e.g. CD4-FITC).

☒ The axis scales are clearly visible. Include numbers along axes only for bottom left plot of group (a 'group' is an analysis of identical markers).

☒ All plots are contour plots with outliers or pseudocolor plots.

☒ A numerical value for number of cells or percentage (with statistics) is provided.

Methodology

Sample preparation	Large intestines were dissected, fat tissues were removed. Intestines were cut open longitudinally and washed in PBS. Intestines were then cut into 3 cm-long pieces, washed, and shaken in PBS containing 1mM dithiothreitol (DTT) for 10min at RT (room temperature). Intestines were incubated with shaking in PBS containing 30mM Ethylenediaminetetraacetic acid (EDTA) and 10mM HEPES at 37°C for 10min for two cycles. The tissues were then digested in the RPMI 1640 medium (Thermo Fisher Scientific) containing DNase I (150ug/ml, Sigma) and collagenase VIII (200U/ml, Sigma) at 37°C in a 5% CO2 incubator for 1.5h. The digested tissues were homogenized by vigorous shaking and passed through 100 um cell strainer. Mononuclear cells were then harvested from the interphase of an 80% and 40% Percoll gradient after a spin at 2500rpm for 20min at RT.<br>For isolation of mononuclear cells from lung, tissues were dissected and blood clots, fat tissues, and bronchus were discarded. Lung tissues were cut into pieces and digested with 5ml RPMI 1640 medium (Thermo Fisher Scientific) containing DNase I (75ug/ml, Sigma) and collagenase VIII (250U/ml, Sigma-Aldrich) at 37°C for 40 min. The digested tissues were homogenized by vigorous shaking and passed through at 70um cell strainer. Mononuclear cells were then harvested from the interphase of a 40 and 80% Percoll gradient after a spin at 2500rpm for 20min at RT.<br>For isolation of mononuclear cells from fat, gonadal adipose tissues were dissected from male mouse. Gonadal adipose tissues were cut into small pieces and then digested in 4 ml RPMI 1640 medium containing collagenase II (400U/ml, Sigma) at 37°C for 20 min. The digested tissues were homogenized by vigorous shaking and passed through 100 um cell strainer. Mononuclear cells were then harvested after a spin at 2000rpm for 10min at RT.<br>For isolation of mononuclear cells from liver, tissues were dissected and mechanically disrupted followed by passing through a 100 um nylon mesh. Cell suspension was spin at 40g for 3min and supernatant was harvested. Mononuclear cells were then harvested from the interphase of a 40 and 80% Percoll gradient after a spin at 2500rpm for 20min at RT.<br>Cell suspensions were prepared from the thymus, spleen, inguinal lymph nodes and mesenteric lymph nodes by gentle mechanical disruption followed by passing through a 50 um nylon mesh.
Instrument	Gallios flow cytometer (Beckman Coulter); BD FACSAria™ III sorter (BD biosciences); MoFlo Astrios™ sorter (Beckman Coulter)
Software	Gallios flow cytometer was used for collecting flow data and Flowjo_ V10 software was used for analyzing flow data .
Cell population abundance	About 1 million Tregs were got from the spleen from 3 mice, and the purity of the samples was about 98% as determined by flow cytometry.
Gating strategy	In all experiments, populations were gated on FSC/SSC. Dead cells were excluded by using LIVE/DEAD™ Fixable Violet Dead Cell Stain Kit. Cells populations were identified as described in Figures or Figure legends. Positive and negative cells were identified based on clear boundaries between the two populations. For markers with no clear boundaries between positive and negative populations, second antibody only was used as a control.

[x] Tick this box to confirm that a figure exemplifying the gating strategy is provided in the Supplementary Information.

Magnetic resonance imaging

Experimental design

Design type	*Indicate task or resting state; event-related or block design.*
Design specifications	*Specify the number of blocks, trials or experimental units per session and/or subject, and specify the length of each trial or block (if trials are blocked) and interval between trials.*
Behavioral performance measures	*State number and/or type of variables recorded (e.g. correct button press, response time) and what statistics were used to establish that the subjects were performing the task as expected (e.g. mean, range, and/or standard deviation across subjects).*

Acquisition

Imaging type(s)	*Specify: functional, structural, diffusion, perfusion.*
Field strength	*Specify in Tesla*
Sequence & imaging parameters	*Specify the pulse sequence type (gradient echo, spin echo, etc.), imaging type (EPI, spiral, etc.), field of view, matrix size, slice thickness, orientation and TE/TR/flip angle.*
Area of acquisition	*State whether a whole brain scan was used OR define the area of acquisition, describing how the region was determined.*

Diffusion MRI [ ] Used [ ] Not used

Preprocessing

Preprocessing software	*Provide detail on software version and revision number and on specific parameters (model/functions, brain extraction, segmentation, smoothing kernel size, etc.).*

Normalization	*If data were normalized/standardized, describe the approach(es): specify linear or non-linear and define image types used for transformation OR indicate that data were not normalized and explain rationale for lack of normalization.*
Normalization template	*Describe the template used for normalization/transformation, specifying subject space or group standardized space (e.g. original Talairach, MNI305, ICBM152) OR indicate that the data were not normalized.*
Noise and artifact removal	*Describe your procedure(s) for artifact and structured noise removal, specifying motion parameters, tissue signals and physiological signals (heart rate, respiration).*
Volume censoring	*Define your software and/or method and criteria for volume censoring, and state the extent of such censoring.*

Statistical modeling & inference

Model type and settings	*Specify type (mass univariate, multivariate, RSA, predictive, etc.) and describe essential details of the model at the first and second levels (e.g. fixed, random or mixed effects; drift or auto-correlation).*
Effect(s) tested	*Define precise effect in terms of the task or stimulus conditions instead of psychological concepts and indicate whether ANOVA or factorial designs were used.*

Specify type of analysis: ☐ Whole brain ☐ ROI-based ☐ Both

Statistic type for inference (See Eklund et al. 2016)	*Specify voxel-wise or cluster-wise and report all relevant parameters for cluster-wise methods.*
Correction	*Describe the type of correction and how it is obtained for multiple comparisons (e.g. FWE, FDR, permutation or Monte Carlo).*

Models & analysis

n/a	Involved in the study
☐	☐ Functional and/or effective connectivity
☐	☐ Graph analysis
☐	☐ Multivariate modeling or predictive analysis

Functional and/or effective connectivity	*Report the measures of dependence used and the model details (e.g. Pearson correlation, partial correlation, mutual information).*
Graph analysis	*Report the dependent variable and connectivity measure, specifying weighted graph or binarized graph, subject- or group-level, and the global and/or node summaries used (e.g. clustering coefficient, efficiency, etc.).*
Multivariate modeling and predictive analysis	*Specify independent variables, features extraction and dimension reduction, model, training and evaluation metrics.*
